# Human thymoma-associated mutation of the GTF2I transcription factor impairs thymic epithelial progenitor differentiation in mice

**DOI:** 10.1038/s42003-022-04002-7

**Published:** 2022-09-29

**Authors:** Orlando B. Giorgetti, Anja Nusser, Thomas Boehm

**Affiliations:** 1 Department of Developmental Immunology, Max Planck-Institute of Immunobiology and Epigenetics, Stuebeweg 51, D-79108 Freiburg, Germany; 2 Faculty of Medicine, University of Freiburg, Breisacher Str. 153, D-79110 Freiburg, Germany

**Keywords:** Immunology, Cancer, Genetics

## Abstract

Few human tumours present with a recurrent pathognomonic mutation in a transcription factor. Thymomas are an exception, with the majority of some subtypes exhibiting a distinct somatically acquired missense mutation in the general transcription factor GTF2I. Co-dominant expression of wild-type and mutated forms of Gtf2i in the mouse thymic epithelium is associated with aberrant thymic architecture and reduced thymopoietic activity. Phenotypic and molecular characterization of the mutant epithelium indicates that medullary differentiation is particularly affected as a result of impaired differentiation of bi-potent epithelial progenitors. The resulting gene expression signature is dominated by that of immature cortex-like thymic epithelial cells. TCR repertoire analysis of the cytopenic T cell compartment indicates efficient intrathymic selection; hence, despite marked homeostatic proliferation of T cell clones, autoimmunity is not observed. Thus, our transgenic mouse model recapitulates some aspects of the pathophysiology of a genetically defined type of human thymoma.

## Abstract (web summary)

Thymic architecture and T cell repertoire analysis of a mouse model for thymoma and the role of the transcription factor GTF2I shows suitability of this model to recapitulate human thymomas and a severe effect of Gtf2i mutations on the medullary compartment.

## Introduction

Thymomas are rare tumours arising from the thymic epithelium, presenting as mediastinal masses. Schemes for classification and clinical staging are the WHO^1^, Masaoka-Koga^2^, and TNM^3^ protocols. They distinguish several subtypes of thymomas with variable degrees of malignant potential; thymic carcinomas are also recognized as distinct entities. Certain thymoma subtypes are associated with a high prevalence of autoimmune syndromes, most notably myasthenia gravis^4^, suggesting that the neoplastic microenvironment perturbs the generation of a self-tolerant T cell repertoire^5^. The molecular characterization of human thymomas and thymic carcinomas is hampered by their complex histoarchitecture^1–3^ and the dynamic age-dependent activity of the thymus^6^. The prognosis of thymomas and thymic carcinomas depends on the age of the patient, the extent of the disease, the particular histological subtype, and the extent of surgical resection^1–3^.

The discovery of a unique recurrent mutation in the gene encoding the general transcription factor GTF2I in thymomas of the A and AB subtypes^7^ (but also in a small fraction of B subtypes^8^) represents a major step forward towards the molecular characterization of these tumours; the mutation invariably affects leucine (L) residue 424, which is always converted to histidine (H), suggesting that this p.L424H missense mutation has tumour-promoting properties^7^. This recurrent signature is even more remarkable since thymomas in general carry one of the lowest mutational burdens among human tumours^9^. The development of targeted therapies directed against the pathophysiological consequences of this tumour-specific lesion would clearly benefit from the availability of suitable animal models that recapitulate at least some aspects of the unique histopathological changes in the mutant thymic microenvironment. To the best of our knowledge, no such animal model has yet been described. Here, we describe the generation of a mouse model exhibiting tissue-specific expression of the mutant form of the mouse orthologue of the human GTF2I gene to answer two questions. First, does the mutated form of Gtf2i affect the differentiation of mouse thymic epithelial cells in vivo? Second, if so, does the mutated Gtf2i differentially affect cortical and medullary compartments? Encouragingly, the phenotype of our transgenic mice resembles some aspects of human thymomas, indicating that mice are a suitable model species to assess the biology of thymomas associated with *Gtf2i* mutations. Moreover, we demonstrate that the medullary compartment of the thymic epithelium is more severely affected than the cortex. Our results encourage the future development of additional mutant mouse models incorporating the facility of mosaic expression of mutant Gtf2i in a subset of thymic epithelial cells.

## Results

### Expression of the mutated form of Gtf2i in thymic epithelial cells

We expressed the mutated form of the Gtf2i transcription factor under the control of the mouse *Foxn1* promotor (see Methods). We chose to express the delta variant of *Gtf2i*, since previous work has shown that this isoform is responsive to growth factor signalling^10^, and that Fgf signals are important for the expansion of the thymic epithelial microenvironment^11^. Residue L424 in the longest human and mouse isoforms corresponds to residue L384 in the Gtf2i delta isoform (Genbank accession number AK147201.1)(Supplementary Fig. 1a). The *Foxn1* gene promoter was chosen, because *Foxn1* encodes an evolutionarily conserved transcription factor^12^ that is expressed in all thymic epithelial cells (TECs) and is essential for their differentiation and maturation^13,14^. In our transgenic mice, the mutated form of *Gtf2i* is expressed in addition to the wild-type gene at approximately similar levels (Supplementary Fig. 1b–d). The transgenic mice are viable, fertile, and do not exhibit obvious behavioural changes. No excess deaths occurred during the observation period of one year. Mild hyperkeratosis of the skin (where the *Foxn1* promoter is also active^14^) was associated with occasional necrosis of the tip of the tail. Importantly, neither overt autoimmune symptoms nor increased susceptibility to infection were observed. Despite the unremarkable clinical appearance, several abnormalities in the immune system were recorded, as described below.

### Cytological phenotype of transgenic thymic epithelial cells

**Fig. 1:**
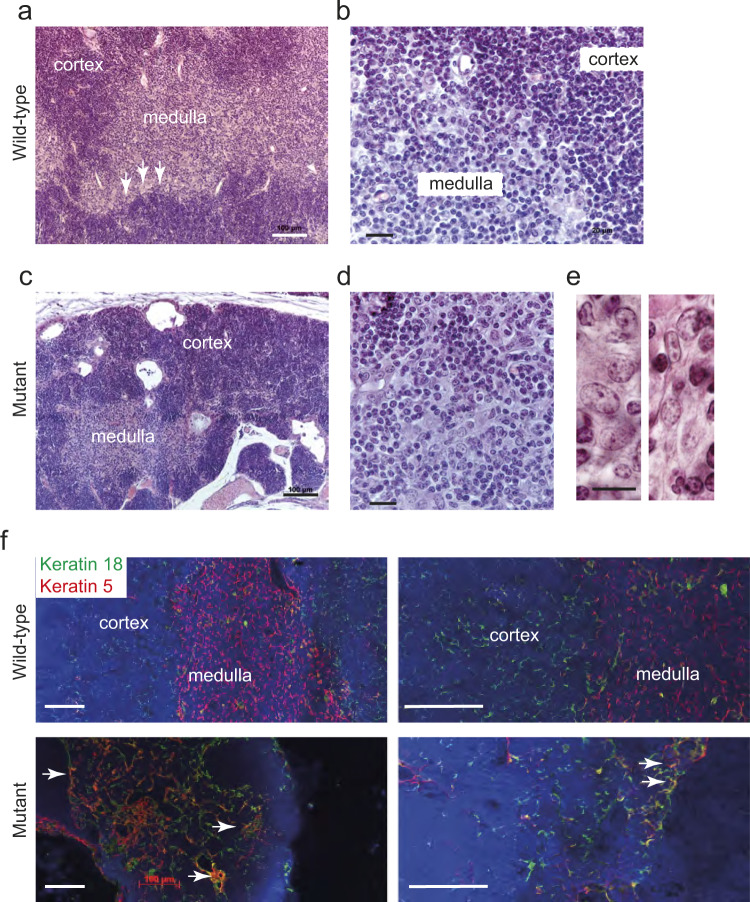
Tissue structure of thymi in *Gtf2i* mutant mice. **a** The wild-type thymus is characterized by well-demarcated cortical and medullary regions, and a sharp cortico-medullary junction; the cortex is densely packed with lymphocytes. Scale bar, 0.1 mm. **b** Higher magnification of wild-type thymus highlights the sharp transition of cortex and medulla. Scale bar, 0.02 mm. **c** The mutant thymus has an irregular structure, exhibits numerous cysts, particularly in the cortex, and a smaller medullary area. Scale bar, 0.1 mm. **d** Higher magnification of the mutant thymus highlights the presence of small lymphocyte-rich islands. Scale bar, 0.02 mm. **e** High-power view of epithelial islands in the mutant thymus indicates the presence of both ovoid epithelium (pale round nuclei, occasionally with prominent nucleoli; left panel) and spindle epithelial cells (pale elongated nuclei). Scale bar, 0.01 mm. **f** Immunohistochemical characterization of thymi using antibodies directed against keratin 5 (red), and keratin 18 (green), demarcating medullary and cortical areas, respectively. The mutant thymus is characterized by numerous keratin 5/keratin 18-double-positive cells (yellow; arrows), indicative of an immature epithelial phenotype. Scale bars, 0.1 mm.

**Fig. 2:**
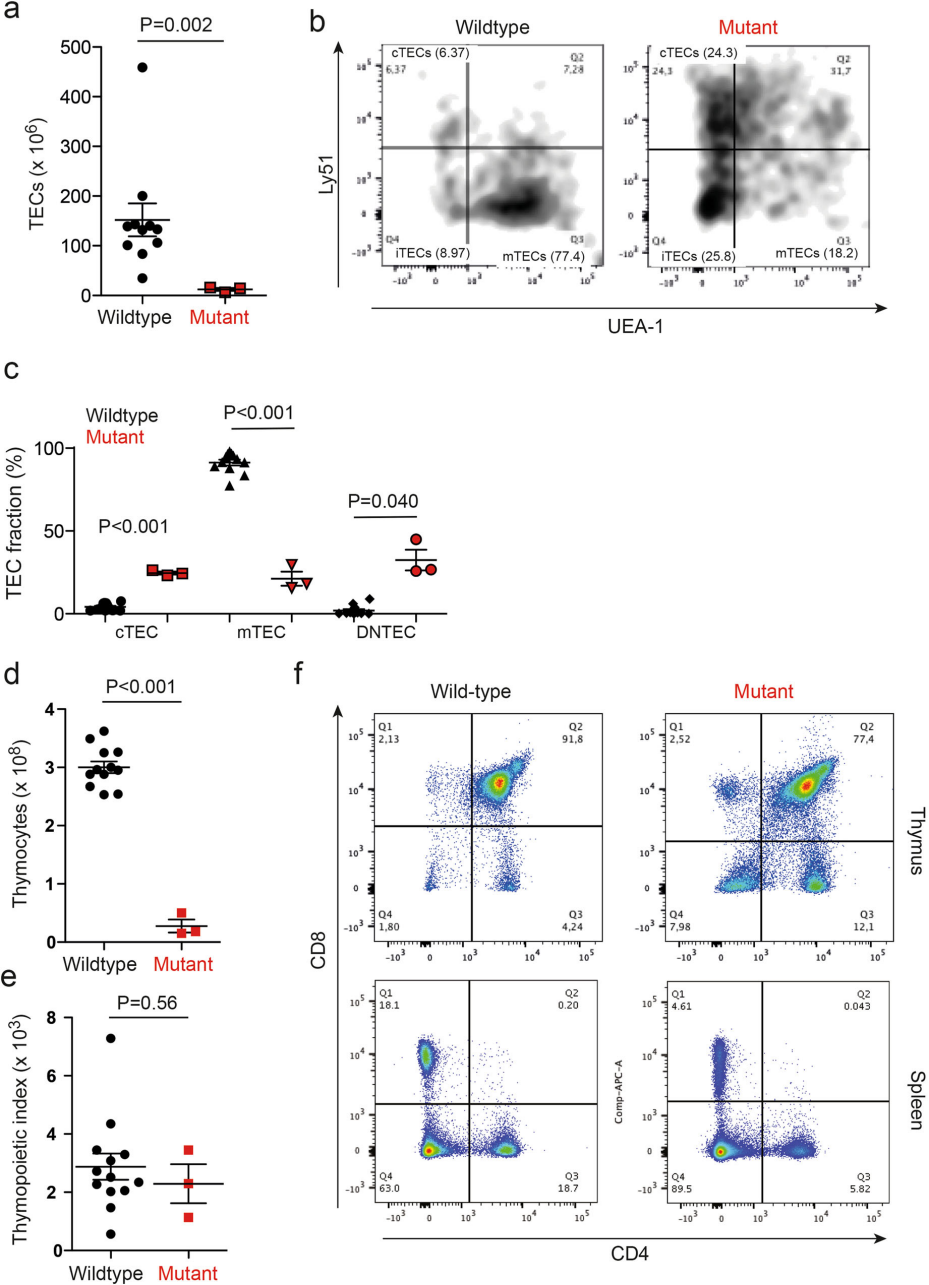
Characterization of mutant thymopoiesis. **a** Enumeration of TECs in wild-type and mutant thymi. **b** Flow cytometric analysis using anti-Ly51 antibodies, and UEA-1 lectin. Ly51^+^UEA-1^–^ cortical TECs (cTECs) are found in the upper left quadrant; Ly51^–^UEA-1^+^ medullary TECs (mTECs) are found in the lower right quadrant; Ly51^–^UEA-1^–^ immature TECs (iTECs) are found in the lower left quadrant. The percentages of cells in the respective quadrants are indicated. **c** Enumeration of TEC subsets in indicated gates (cTEC, cortical TECs; mTEC, medullary TECs; iTEC, immature TECs). t-test, two-sided. **d** Enumeration of CD45^+^ thymocytes. **e** Thymopoeitic index as a measure of thymopoietic capacity calculated as the number of thymocytes divided by the number of TECs. **f** Representative flow cytometric profiles of thymocytes and splenocytes stained with anti-CD4 and anti-CD8 antibodies.

The effect of the *Gtf2i* mutation on thymic epithelial cells (TECs) was determined at several levels at 4-6 weeks of age. Upon gross inspection, the transgenic thymus is located in the upper mediastinum. Compared to the wild-type thymus (Fig. 1a, b) the mutant thymus is smaller, and exhibits numerous cysts associated with an irregular arrangement of cortical and medullary regions (Fig. 1c–e). Immunohistochemical analysis with antibodies directed against keratin 5 (identifying medullary epithelium) and keratin 18 (identifying cortical epithelium) revealed numerous double-positive epithelial cells (Fig. 1f); this epithelial phenotype is characteristic of undifferentiated thymic epithelium^14,15^. In accordance with the small size of thymi, the number of TECs is several-fold lower in mutant mice (Fig. 2a). Flow cytometric studies of enzymatically dissociated CD45^–^EpCAM^+^ epithelial cells revealed a paucity of Ly51^–^UEA-1^+^ medullary thymic epithelial cells (mTECs), which dominate the TEC population in the wild-type thymus at this age; instead, the mutant thymus contains a large fraction of Ly51^–^UEA-1^–^ double-negative TECs (Fig. 2b, c). The presence of Ly51^–^UEA-1^–^ TECs was accompanied by an increased fraction of Ly51^+^UEA-1^–^ cells that resemble the cortical phenotype (Fig. 2b, c). Collectively, the morphological characteristics of mutant TECs are indicative of an undifferentiated state, resembling the TEC compartment of the embryonic thymus in late gestation^16^. We conclude that the presence of the *Gtf2i* mutation causes an incomplete block of TEC differentiation, leading to an accumulation of immature TECs.

### Thymopoietic activity of the mutant thymus

The morphological and molecular analyses described above converge to indicate that the TEC compartment in mutant mice is in an immature state. This phenotype is accompanied by reduced thymopoietic activity of the mutant thymus, which results in a drastically reduced number of thymocytes (Fig. 2d); however, since the number of TECs is also diminished, the ratio of thymocytes to TECs is similar to the wild-type mouse (Fig. 2e). The thymocyte compartment is characterized by a diminished proportion of CD4^+^CD8^+^ double-positive cells and an increased CD4^+^/CD8^+^ ratio (Fig. 2f). T cell cytopenia persists in the periphery (Fig. 2f). We attribute the reduced generative capacity of the thymus to the disorganized epithelial microenvironment.

### Expression pattern of *Gtf2i* in TEC progenitor populations

**Fig. 3:**
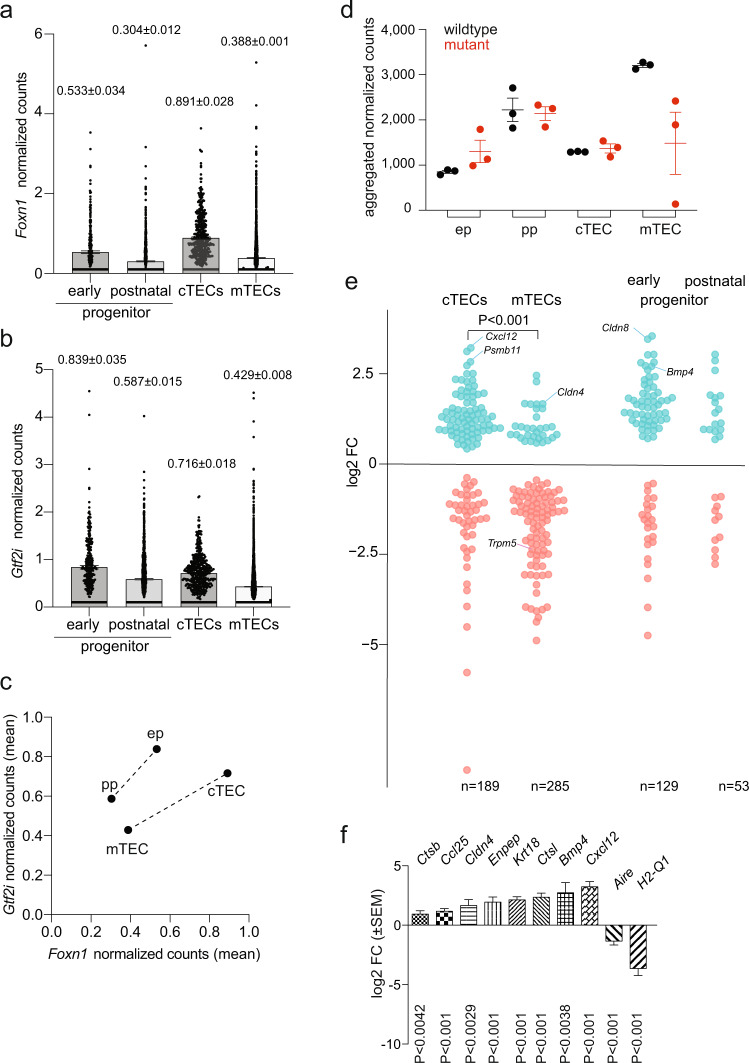
Molecular phenotype of mutant thymic epithelium. **a** Expression of *Foxn1* in indicated TEC compartments expressed as aggregate normalised counts. Mean±s.e.m. In cTECs, ~ 90% of all cells express *Foxn1*; in mTECs, this figure is ~50% (Supplementary Data 6). **b** Expression of *Gtf2i* in indicated TEC compartments expressed as aggregate normalised counts. Mean±s.e.m. **c** Correlation of *Gtf2i* and *Foxn1* expression levels in indicated TEC compartments. The mean values shown in **a**, and **b** are used. **d** Aggregated normalised transcript counts in wildtype and mutant TECs for the four relevant compartments. ep, early progenitor; pp, postnatal progenitor; cTEC, cortical TECs; mTEC, medullary TECs. Mean±s.e.m.; each dot represents one mouse. **e** Differential gene expression of compartment-specific gene sets in the indicated TECs of mutant mice. Each dot represents an individual gene; the number of genes assigned to the gene sets are indicated at the bottom. Some of the genes discussed in the text are marked. **f** Expression levels of select genes cTEC- and mTEC-associated genes as determined by RNA-seq. Mean±s.e.m. (see text or details).

In order to identify the TEC population(s) most susceptible to the *Gtf2i* mutation, we first compared the expression patterns of *Foxn1* (Fig. 3a) and *Gtf2i* (Fig. 3b and Supplementary Fig. 2) in the different populations of TECs, which we have previously characterised by scRNA-seq^11^. *Foxn1*^11^ and *Gtf2i* (Supplementary Fig. 2) expression levels differ between different clusters of transcriptionally related TECs. At 4 weeks of age, the highest levels of *Gtf2i* were found in the early (embryonic-type) bi-potent progenitor, whose differentiation trajectory is biased towards cortical epithelium; the postnatal bi-potent progenitor population, which preferentially generates mTECs, exhibits somewhat lower expression levels (Fig. 3b). With respect to mature TECs, cTECs tend to have higher levels than mTECs (Fig. 3b). The relative expression levels of *Gtf2i* in the progenitor populations are similar to those of *Foxn1*, which also exhibits higher expression levels in the early progenitor; the same is true for cTECs and mTECs, where *Foxn1* is expressed at higher levels in cTECs (Fig. 3c). Overall, the expression pattern of *Gtf2i* suggests that the mutation should predominantly affect the early progenitor and cTEC compartments. This appears to be the case, since the mutant epithelium is characterized by mal-differentiated cTEC-like cells associated with a paucity of mTECs (Figs. 1 and 2).

### Molecular signature of mutant thymic epithelial cells

In order to more precisely characterise the molecular phenotype of mutant epithelium, we turned to our previous work^11^, which assigned four largely non-overlapping gene sets to mark two bi-potent progenitor populations, and mature cTEC and mTEC populations, respectively. To this end, we purified TECs by flow cytometry as CD45^–^EpCAM^+^ cells and subjected them to differential gene expression analysis by RNA-seq. As expected from the abnormal phenotype of mutant TECs, a substantial number of differentially regulated genes were detectable (Supplementary Data 1). Next, we examined the expression levels of genes associated with each of the aforementioned four sets of unique cell populations. With respect to the early progenitor gene set, the results indicate that about two thirds of these genes are differentially regulated, and twice as many genes are upregulated than downregulated (Fig. 3d); the genes associated with the postnatal progenitor subset follow a similar pattern (Fig. 3d). Impaired differentiation of the mTEC compartment and preponderance of cTEC-like cells detected by cytological analyses is mirrored in the effects on up- and downregulated genes sets for mature TEC types (Fig. 3d). When plotting aggregated normalized counts for the gene sets of the four TEC compartments, the difference between wild-type and mutant epithelium is most notable for mTECs (Fig. 3e), although the degree of mTEC perturbation varies. Turning to individual genes, we observed the following. The expression levels of *Bmp4*, which is expressed in immature TECs and hence whose expression peaks in the embryonic thymus^17,18^, are increased about 6-fold, supporting the notion of a general immaturity of the TEC compartment (Fig. 3f). Other genes, whose expression marks cTECs, are upregulated 4-8 fold in mutant TECs, such as *Cxcl12*, encoding the ligand for the Cxcr4 chemokine receptor expressed on haematopoietic precursors entering the thymus^19,20^, or *Ctsl* (cathepsin L), which is required for maturation of MHCII molecules and selection of CD4 cells^21^ (Fig. 3f); the latter observation may explain, at least in part, the skewed intrathymic CD4/CD8 ratio (Fig. 2f). Other cTEC-specific genes^22^, such as *Krt18*, *Enpep* (encoding the Ly51 cell surface marker), and *Ccl25* (encoding an important chemoattractant of cortical epithelium), are increased about 2 to 4-fold relative to the wild-type TEC compartment (Fig. 3f). This aspect of the molecular phenotype is compatible with the overrepresentation of cTEC-like cells in the epithelium (Fig. 2b, c). By contrast, expression levels of medullary marker genes^23^ exhibit notable reductions. For instance, *Mhc(H2-Q1)* and *Aire*, are expressed at lower levels than in wild-types (Fig. 3f), reflecting the paucity of Ly51^–^UEA-1^+^ mTECs (Fig. 2b, c); the same is true for reduced levels of *Trmp5* (Fig. 3d), which is characteristically expressed in the medullary tuft cells^24^, which are a post-*Foxn1*/post-*Aire* lineage of thymic epithelium^11^. Of note, the expression of *Cldn4* (Claudin 4), which has been associated with a medullary precursor phenotype^25^ is increased, in line with the block in TEC differentiation (Fig. 2b, c). Despite the complex changes in the transcriptome of mutant TECs, *Gtf2i*^L384H^-expressing epithelia maintain their cellular identity, as indicated by unchanged *Foxn1* expression levels (Supplementary Data 1).

In order to gain a more comprehensive view of changes in the transcriptome of mutant thymic epithelium, we subjected the RNA-seq data to pathway analysis; Supplementary Data 2 and 3 report the results for up- and downregulated genes, respectively. This study was motivated by previous work introducing diagnostic expression signatures for thymoma subtypes^26^, and reports of transcriptome changes in epithelial cells expressing mutated GTF2I^27^. In our ex vivo population of TECs, GO terms associated with ATP production (such as GO:0009060) are enriched among the genes up-regulated in mutant TECs, in line with previous findings of increased glycolysis in cultured cells expressing the mutant GTF2I transcription factor^27^. Enrichment of GO terms associated with the regulation of apoptosis (such as GO:2001243, and GO:2001235) indicate profound changes in the regulation of cell death; the gene expression profiles suggest that negative regulators of the intrinsic apoptotic pathway dominated the mutant transcriptional landscape (summarized in Supplementary Data 4), compatible with previous work in cell lines^27^. The same is true for genes associated with the regulation of the cell cycle (GO:0007346) and DNA repair (GO:0006281). However, some discrepancies between the expression profiles of cultured cells and ex vivo TECs are recognizable; for instance, changes in the expression levels of genes associated with epithelial-mesenchymal transition are variable (*Snai*1, log2 fold-change −5.02; *Snai2*, +1.89; *Vim*, −1.60), whereas they were shown to be uniformly upregulated in tissue culture^27^.

Most notable among the enriched pathways in down-regulated genes of the mutant TEC compartment are GO terms associated with neuronal development (GO:0007409), embryonic organ development (GO:0048562), and WNT signalling (GO:0060828). Among the down-regulated genes associated with WNT signalling (Supplementary Data 4), we note that previous in vivo studies are in support of our findings; loss of Kremen1 blocks TEC differentiation^28^, and Notch1 signalling is required for the development of the medulla^29,30^. Some components of the SHH signalling pathway are downregulated in the mutant TECs (*Shh*, log2 fold-change −8.03; *Dhh*, −0.98; *Ptch1*, −1.99; *Ptch2*, −7.49) (Supplementary Data 1). These findings stand in contrast to results reported for human tumour samples, in which genes associated with these pathways were found to be up-regulated^26^. In sum, our studies suggest that the presence of the mutant Gtf2i transcription factor blocks TEC differentiation and increases the survival of the immature epithelium.

### Histopathological classification of TEC compartment

**Fig. 4:**
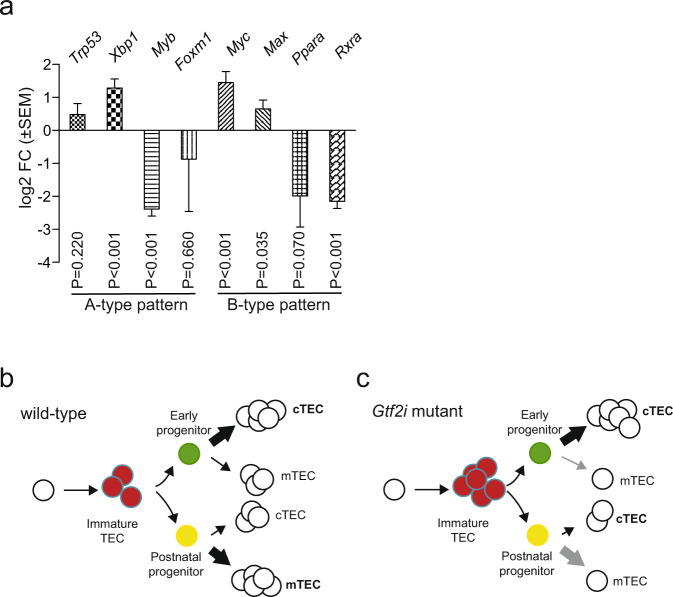
Molecular classification of mutant thymic epithelium. **a** Expression levels of select genes as determined by RNA-seq. Mean±s.e.m. Qualitative expression patterns are indicated for A-type and B-type human thymomas, indicating that the mouse models do not conform to one human entity. **b** Schematic description of differentiation trajectories in wild-types to cTECs and mTECs from immature TECs via bipotent early and postnatal progenitors, each with a distinct differentiation bias^11^. **c** Perturbed differentiation trajectories in the mutant epithelium, indicating an accumulation of immature TECs and an impaired differentiation capacity of bi-potent progenitors.

The phenotypic appearance of the mutant thymus exhibits characteristics that are typically associated with human A-type, AB-type, and B-type thymomas. For instance, the extended reticular nature of wild-type cortical cells is less pronounced in the mutant mice (Fig. 1), reminiscent of the spindle-like nature of the epithelium in A-type and AB-type thymomas^1^; however, small regions of ovoid epithelial cells with only few lymphocytes were also seen (Fig. 1). The disorganized cortex of the mutant thymus is accompanied by a malformed medulla (Fig. 1), and thus resembles a type B thymoma more than a type A thymoma^1^. We conclude that the mutant microenvironment is of mosaic nature and exhibits regional diversification associated with features of all three recognized thymoma types. The mixed histopathological phenotype is reflected in the RNA expression data. For instance, the overexpression of *Cstb* (Cathepsin B) observed here (Fig. 3f) is also seen in type A and type AB thymomas in humans^31^. A recent study has found that human thymomas can be classified using the expression levels of a select number of genes^26^. Human Type A thymomas express high levels of *TRP53*, *XBP1*, and reduced levels of *MYC*, *MAX*, *MYB*, and *FOXM1* genes. By contrast, AB-like thymomas exhibit increased levels of *MYB*, and *FOXM1* genes associated by reduced levels of *TRP53*; B-type thymomas possess low levels of *TRP53*, *PPARA*, *RXRA*, and *XBP1* genes, and increased levels of *MYC*, *MAX*, and *MYB*^26^. The mutant TECs in our transgenic mice exhibit a gene expression pattern that resembles thymomas of either A-type (elevated levels of *Trp53*, *Xbp1*; reduced levels of *Myb*, *Foxm1*) or B-type (elevated levels of *Myc*, *Max*; reduced levels of *Ppara*, *Rxra*) (Fig. 4a); whether this mixed expression pattern is a result of species differences or a consequence of the uniform expression of the mutated *Gtf2i* gene in all TECs, rather than in a patchy form in the human tumours, is currently unclear. In sum, the phenotype of mouse TECs expressing the mutant *Gtf2i* gene is best characterized as an A type-like thymoma, although some features of human AB-type and B-type thymomas are also present. Collectively, our results indicate that the presence of the *Gtf2i* mutation perturbs the differentiation of the epithelial compartment, which is generated by the activity of two types of bi-potent progenitors (Fig. 4b)^11^. Co-dominant expression of wild-type and mutant Gtf2i most prominently affects the emergence of mTECs, resulting in a microenvironment that is dominated by cTECs (Fig. 4c); in addition, the presence of a large fraction of seemingly undifferentiated Ly51^–^UEA-1^–^ TECs suggests an accumulation of progenitor cells.

### T cell receptor repertoires

**Fig. 5:**
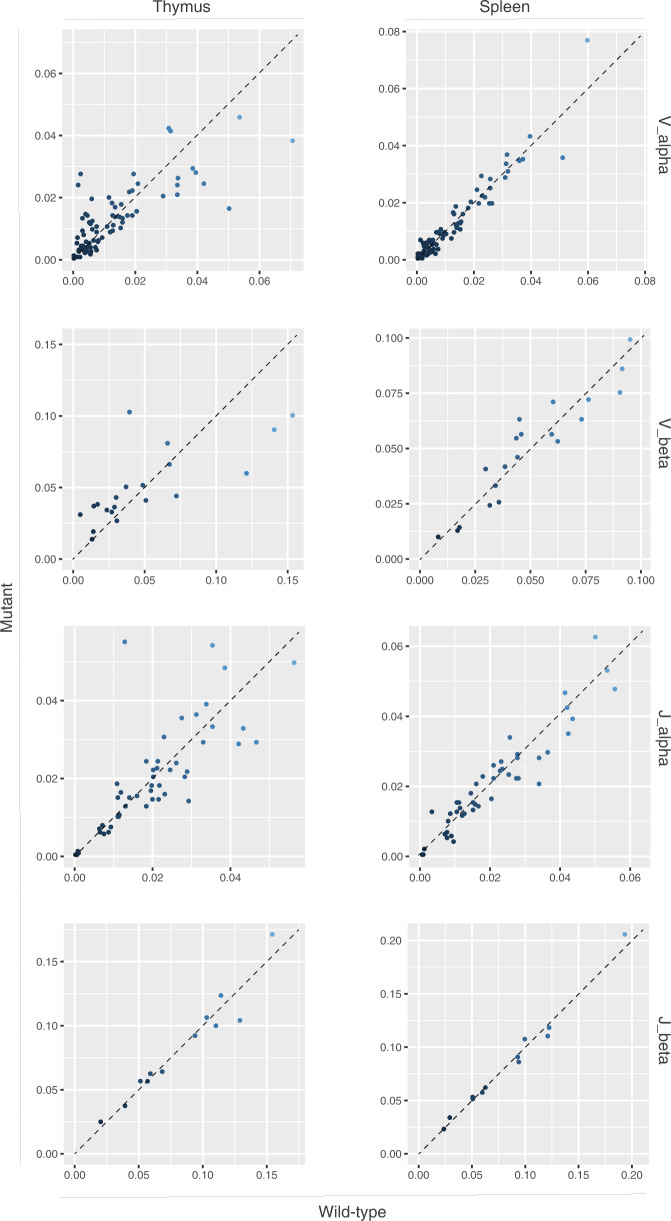
Usage of TCR alpha and beta chains in thymus and spleen. The fractions of clonotypes containing V and J elements are compared between wild-type (x axis) and mutant (y axis) repertoires. The dotted line indicates the line of perfect correspondence.

**Fig. 6:**
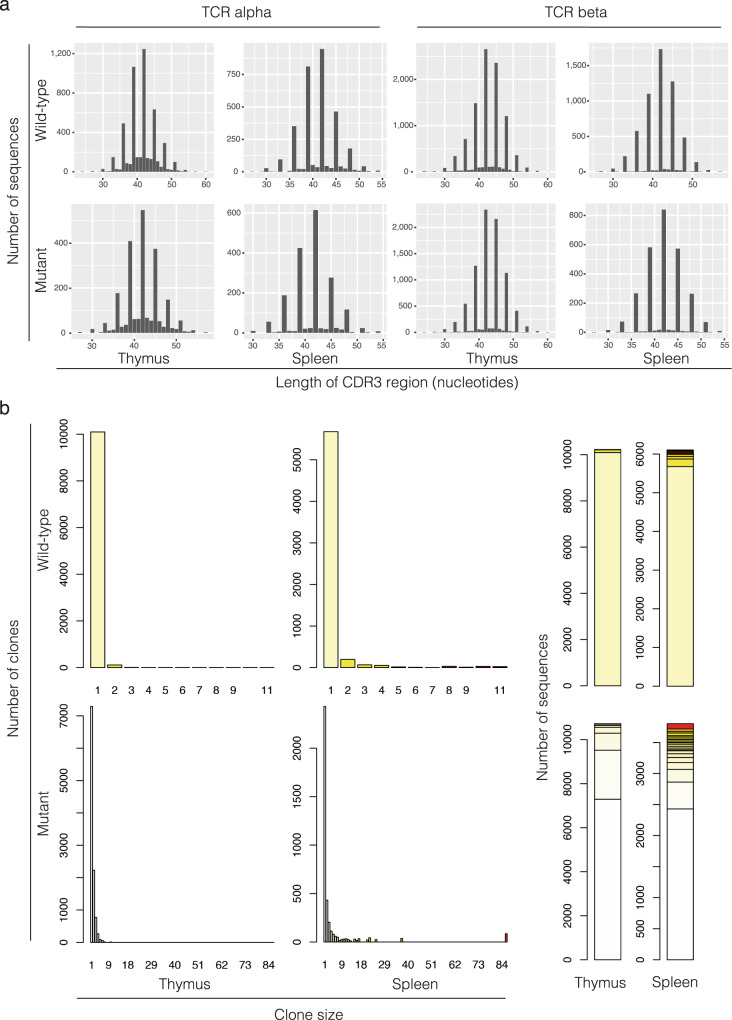
Sequence composition of TCR alpha and beta chains. **a** Length distributions of CDR3 regions. Note the peaks occurring at every third nucleotide position, representing in-frame sequences; CDR3 sequences of other sizes (indicative of out-of-frame sequences) make only minor contributions to the overall repertoire. The latter type is essentially lacking in sequences of the peripheral repertoire (spleen), as a result of efficient purging of non-functional rearrangements from the repertoire. **b** Clone sizes of TCR beta clonotypes. Note that the wild-type exhibits a larger number of clones that are associated with only a single UMI, indicative of a greater complexity of the repertoire.

Next, we considered the possibility that despite the lack of overt autoimmunity, the mal-differentiated TEC compartment might lead to aberrations in the selection process of the T cell repertoire. To this end, we established datasets of full-length *Tcra* and *Tcrb* sequences by multiplex amplification of cDNAs barcoded with unique molecular identifiers (UMIs)^32^. Initially, we compared the usage of V and J elements in the assembled *Tcra* and *Tcrb* genes as a measure of changes in the accessibility of the antigen receptor loci. No drastic changes in the usage of V and J elements of both chains are detectable in the mutant thymus and in the peripheral T cell pool (Fig. 5). The large collections of clonotype sequences afforded us the possibility to evaluate the efficiency of the intra-thymic selection process. To this end, we examined the fractions of in-frame and out-of-frame *Tcra* and *Tcrb* sequences in the thymus and compared them to those in the peripheral T cell populations of the spleen. In both wild-type and mutant animals, the proportion of in-frame sequences significantly increases in the peripheral repertoire. This indicates that non-functional T cell receptors are efficiently purged during the selection in the thymus (Fig. 6a). Collectively, these results suggest that, despite the abnormal thymic microenvironment, the generation and selection of the TCR repertoire occurs normally. Hence, even in the presence of peripheral T cell lymphopenia in the mutant animals (Fig. 2f), the diversity of their T cell receptor repertoires appears to be sufficient for adequate immune surveillance and protection, at least under the conditions of low antigen exposure and infection pressure in an animal facility.

As a further measure of adequate T cell function, we examined the repertoire structure in the thymus and the spleen. This analysis rests on the fact that each mRNA molecule is unique-molecular-identifier (UMI)-tagged during cDNA synthesis; thus, the number of different UMIs associated with a given clonotype in the transcriptome represents a measure of clonotype abundance in the repertoire. We consider clonotype abundance as a proxy for cell number. In the thymus of wild-type mice, more *Tcrb* clonotypes are associated with one or two UMIs than in the mutants; this difference can be attributed to the great diversity of the naïve T cell receptor repertoire in the wild-type, and lymphopenia in the mutant, respectively (Fig. 6b). Although the peripheral repertoires of both wild-type and mutant mice exhibit evidence of clonal expansions, as indicated by the appearance of high-frequency clonotypes, the repertoire of mutants is far more restricted than that of wild-types. In the wild-type repertoire almost 90% of all sequences are associated with one UMI; however, this is true for only 65% of sequences in the mutant repertoire, indicative of homeostatic proliferation of a restricted repertoire (Fig. 6b).

## Discussion

Here, we describe the first mouse model of human thymomas carrying the equivalent of the GTF2I^L424H^ mutation^7,8^. Our results show that the *Gtf2i*^L384H^ mutation behaves as a dominant trait and that it causes an incomplete block of TEC differentiation. This leads to a preponderance of immature TECs and impaired T cell development. Thus, the phenotype of mutant mice observed here resembles some aspects of the clinical picture of the human disease.

From a histopathological standpoint, the thymus in thymoma patients is thought to represent a mosaic comprised of areas with wild-type microenvironment and adjacent regions dominated by mutant TECs. Most patients presenting with L424H mutation-associated thymoma are adults, compatible with the presumed stochastic nature of the somatic mutation event^7,26^. At present, it is unclear if mutated TECs gain a net numerical advantage over their non-mutated counterparts, or whether they still obey, albeit inefficiently, the intrinsic differentiation programme of thymic epithelia. We speculate that the clinical picture and course of the disease are determined by the type of TEC that experiences the mutational event. For instance, if the mutation affects a TEC with low proliferative potential, the mutation may become physiologically relevant by merely expanding the lifespan of mutant cells, perhaps as a result of a reduced rate of apoptosis. This constellation may lead to thymomas with a low grade of malignancy. Indeed, results of experiments with cell lines suggest that the *GTF2i* mutation is associated with increased survival rates in metabolically unfavourable culture conditions^27^. By contrast, if the mutation afflicts a TEC progenitor cell type^11^, the combination of life-span extension and intrinsic proliferative potential of the target cell might synergize to increase the target cell population and to eventually cause a more aggressive phenotype. This scenario would also explain the occurrence of thymic carcinomas with the *GTF2I* mutation^33^, favoured by the acquisition of additional genetic lesions^27^ The rarity of carcinomas with the *GTF2I* mutation is consistent with the observation that progenitor cells represent only a minor cell population in the TEC compartment^11^; for instance, in the postnatal period of the mouse, the fraction of bi-potent TEC progenitors has been estimated to be in the range of approximately 3%^34^.

In a large multi-omics study of human thymomas, integrative unsupervised clustering was able to recover four molecular subtypes, which segregated well with the WHO histopathological classification scheme^26^. Unfortunately, the mRNA data of the human samples cannot be compared to the RNA-seq results obtained in the present study in a 1:1 fashion. Of note, the human samples were processed without prior purification of TECs^26^, and thus, the cellular composition of the tumour samples is not comparable to our study material. Alternatively, but not mutually exclusively, species-specific differences may explain part of the discrepancies. Nonetheless, we expect that our findings will provide valuable additional information that could be used to further and more specifically evaluate the molecular fingerprint of malignant epithelia in human thymomas.

The discrepancy between the high incidence of autoimmunity in thymoma patients and the lack of such pathology in our mice remains to be explained. Although we cannot rule out the possibility that the lack of immunopathology in our mice is the result of a species-specific difference, we consider it more likely that autoimmunity emerges only when the thymic microenvironment is composed of a mosaic of wild-type and mutant epithelium. Interestingly, recent genome-wide association studies suggested a link between GTF2I and autoimmune disorders, such as primary Sjögren´s syndrome^34^ and others^35–38^. However, most pertinent to the present discussion is the fact that the relevant single nucleotide polymorphisms (SNPs) are situated in non-coding regions of the gene, and that one SNP (rs117026326) is associated with increased expression of GTF2I in salivary gland cells^39^. How these germ-line variants are connected to the phenotypic consequences of the somatically acquired GTF2I^L424H^ mutation is unclear.

The transgenic mice described here should be considered a first-generation mouse thymoma model. It provided the first in vivo evidence that the mutated form of Gtf2i perturbs the differentiation of thymic epithelial cells and indicated that the effects of the Gtf2i mutation are not unique to humans. However, the current implementation does not perfectly recapitulate the human situation. Whereas human thymoma tissue rarely encompasses the entire thymus, so that mutant and normal parts co-exist within the same lobe, all TECs in transgenic mice carry the *Gtf2i* mutation. A second-generation model could be constructed by generating chimaeric mice composed of wild-type and mutant cells in such a way that variable fractions of TECs carry the *Gtf2i* mutation; however, the mutation would be present in the affected cells from the gestational period onwards. Hence, a third-generation version of the *Gtf2i* thymoma model described here should incorporate the facility of conditional induction of the mutation in only a small number of cells; such a strategy was successfully used to activate a wild-type copy of the *Foxn1* gene specifically in single *Foxn1*-deficient precursor cells^40^. In this way, the mutation could be induced at different time points during the lifetime of the mouse to explore whether *Gtf2i* mutations occurring early in life may remain dormant until adulthood, or whether they are more likely to lead to thymic carcinomas.

In conclusion, the results of our study indicate that it is possible to recapitulate at least some aspects of human thymomas in mice. Our transgenic mouse line thus represents a unique starting point for future studies aimed at exploring the cell-intrinsic and cell-extrinsic consequences of a mutant thymic microenvironment in situations that more closely mimic the situations in human thymomas.

## Methods

### Mice

C57BL/6 mice are maintained in the Max Planck Institute of Immunobiology and Epigenetics. The *Foxn1:Gtf2i*^L384H^ transgene was created by inserting a cDNA fragment corresponding to nucleotides 1 to 4300 in GenBank accession number AK147201.1 as a NotI fragment into pAHB14 (ref. ^18^); the TG > AT mutation was inserted into the wild-type cDNA sequence by site-directed mutagenesis according to standard procedures, and verified by Sanger sequencing. Note that L384 is the equivalent of L424 in the human sequence in the delta isoform of Gtf2i. The mutated site creates a recognition sequence for the *Sph*I restriction endonuclease (5′-GC**AT**GC-3′; mutant nucleotides in bold). Transgenic mice were generated on an FVB/N background (FVB/N-tg (Gtf2i^L384H^)1^Tbo^/Mpie) and subsequently backcrossed to a C57BL/6 J background. Mice were kept in the animal facility of the Max Planck Institute of Immunobiology and Epigenetics under specific pathogen-free conditions. Some mutant mice developed necrosis of the tail after about 6 months; this genotype-associated burden precluded an analysis of aged mice. All animals used here were 4-6 weeks old; no sex difference was noted for the thymus phenotype. All animal experiments were performed in accordance with the relevant guidelines and regulations, approved by the review committee of the Max Planck Institute of Immunobiology and Epigenetics and the Regierungspräsidium Freiburg, Germany (licence 35-9185.81/G-15/36).

### Genotyping

Genotyping was carried out using primers XAH163 (5′- GTCCCTAATCCGATGGCTAGCTC, located in the 5′-UTR of the *Foxn1* gene), and XAH474 (5′GTCGGTCTCATAGAGAGCAATGC, located in the *Gtf2i* cDNA sequence). To determine the presence of mutated transcripts, cDNAs were amplified using primers OBG_7 (5′-CACCCACCAAGAGGCTAAAG) and OBG_14 (5′-GAGCCCTTCCACATACAGAAA). The 880 bp amplicon was then digested with *Sph*I, and the resulting fragments resolved by gel electrophoresis; the wild-type form is resistant to digestion, whereas the mutated from gives rise to two fragments, 320 bp and 560 bp.

### Histology

Thymi for hematoxylin/eosin staining were fixed in 4% PFA, embedded in paraffin, and 6 µm sections were stained using standard techniques.

### Immunohistochemistry

Thymi were fixed in 4% PFA, washed in PBS, incubated in 20% sucrose over night and embedded in OCT. 8-10 µm sections were dried over night at room temperature and prior to staining moisturised in PBS followed by a 30 min blocking step (PBS supplemented with 0.5% BSA, 0.2% Tween, antimouse IgG 1:50). Antibody staining was performed at room temperature in staining buffer (PBS supplemented with 0.5% BSA, 0.2% Tween, 3% serum). Sections were stained for 2 h with primary antibodies (ANTI-KERATIN 5, ANTI-KERTAIN 18)), and then for 45 min with secondary antibodies and streptavidin. Sections were washed with PBS between incubations. After staining, sections were mounted in Fluoromount G. Information on antibodies used in this study are given in Supplementary Table 1.

### Image analysis

Images were acquired on Zeiss microscopes (Axioplan 2 or Imager Z1 with ApoTome attachment) equipped with AxioCam MRc 5 cameras.

### Flow cytometry

To generate single cell suspensions for analytical and preparative flow cytometry of TECs, the procedures described by Nagakubo et al.^41^ and Rode et al.^42^ were followed. Relevant staining reagents are listed in Supplementary Table 1. Note that the enzymatic cocktail required to liberate thymic epithelial cells destroys the extracellular domains of CD4 and CD8 surface markers (but not that of the CD45 molecule); hence, when analysis of thymocyte subsets was desired, thymocyte suspensions were prepared in parallel by mechanical liberation, achieved by gently pressing thymic lobes through 40 µm sieves. Cell sorting and analytical flow cytometry were carried out using MoFlow and Fortessa instruments respectively (both from Dako Cytomation-Beckman Coulter); the analysis of flow cytometric experiments was carried out using the FACSDiva Software.

### RT-PCR

RNA was extracted using TriReagent (Sigma, Cat#93289). DNA was removed from RNA extraction usingTURBO DNA-free kit (Invitrogen, Cat#AM1907). RNA was quantified using the Qubit RNA HS Assay Kit (ThermoFisherScientific, Cat#Q32852) and the Qubit 4 Fluorometer (ThermoFisherScientific, Q33226). RNA quality was checked by determining the 18 S/28 S rRNA ratio using the Fragment Analyzer RNA Kit (ThermoScientific, Cat#DNF-471-0500) and the 5200 Fragment Analyzer System (ThermoScientific, Cat#M5310AA). cDNA libraries were prepared from 1 μg of mRNA following poly-A selection using TruSeq stranded mRNA Library Prep (Illumina, Cat#20020595) according to manufacturer’s instructions.

### RNA sequencing and computational analysis of RNA-seq data

RNA-Seq was performed using 3 biological replicas (wild-type) and 4 biological replicas (mutant). The libraries were sequenced in paired-end 75 bp mode at a depth of 25 million reads on an Illumina HiSeq 2500/3000 instrument. Reads were aligned to the reference genome with STAR 2.5.2b-1 (ref. ^43^) and the reference annotation from Ensembl (http://www.ensembl.org/info/data/ftp/index.html). The resulting alignments were quantified at the gene level with featureCounts version 1.6.0.1 (ref. ^44^) and differential expression performed using DESeq2 version 2.11.40.1 (ref. ^45^). The analysis was orchestrated on the in-house version of the Galaxy server based on the Galaxy platform^46^. All tools were used with default parameters. The wild-type data of Swann et al.^47^ were used for comparison. Gene sets were analysed for enriched biological processes using the database for annotation, visualization and integrated discovery (DAVID) version 6.8 Analysis Wizard annotation tool^48,49^.

### TCR sequencing

*cDNA synthesis*. Total RNA from purified TEC were extracted using the TRIzol reagent (Life Technologies, Carlsbad, CA, USA) according to the manufacturer’s protocol. cDNA synthesis was performed using the SMARTScribe Reverse Transcriptase (Clontech, Mountain View, CA, USA) with an oligo-dT primer (5′-AAGCAGTGGTATCAACGCAGAGTTTTTTTTTTTTTTTTTTTTTTTTVN) and SMARTer_Oligo_UMI primer (5′-AAGCAGUGGTAUCAACGCAGAGUNNNNUNNNNUNNNNUCTT[rGrGrGrGrG]) according to the SMARTer RACE 5’RACE protocol (Clontech, Mountain View, CA, USA). The SMARTer_Oligo_UMI is a hybrid primer with riboguanosines representing the last five bases and the remainder representing deoxyribonucleotides, including the U (deoxyuracil); the Ns represent the bar code. The cDNA synthesized was treated with uracil-DNA glycosylase before all reactions from the same individual were combined together. The combined cDNA was purified using the QIAquick PCR Purification Kit (QIAGEN, Hilden, Germany), eluted with 70 μl of diethylpyrocarbonate (DEPC)–treated water, and vacuum-dried. *Library preparation*. The cDNA samples were amplified essentially according to the protocol of Turchaninova et al.^50^, except that Illumina multiplexing primer sequences p5 (5′-ACACTCTTTCCCTACACGACGCTCTTCCGATCT) and p7 (5′-GTGACTGGAGTTCAGACGTGTGCTCTTCCGATCT) were appended to the 5′ ends of their second reaction primers. The first round of PCR amplification was carried out in multiplex manner: 1× Q5 buffer, 0.5 mM deoxynucleoside triphosphate (dNTP), 0.2 μM UPM_S primer (5′-CTAATACGACTCACTATAGGGC), 0.04 μM UPM_L primer (5′-CTAATACGACTCACTATAGGGCAAGCAGTGGTATCAACGCAGAGT), and 0.2 μM of each gene-specific primer (GSP), 2 μl of cDNA, water to 49.5 μl, 0.5 μl of Q5 Hot Start High-Fidelity DNA Polymerase (New England Biolabs); 98 °C for 90 s followed by 23 cycles of 98 °C for 10 s, 65 °C for 20 s, and 72 °C for 45 s, followed by 8-min final extension at 72 °C. GSPs used in the first round were OBG_140 (5′-GGTGCTGTCCTGAGACCGAG) for *Tcra*, and OBG_136 (5′-GATGGCTCAAACAAGGAGACC) for *Tcrb*. Amplicons were size-separated on agarose gels, the region between 500- and 1000-bp excised, and the DNA was extracted using the QIAquick Gel Extraction Kit (QIAGEN) following the protocol provided by the manufacturer (with two PE washes) and lastly eluted in 50 μl of water. For the second round of PCR amplification, each target locus was amplified separately. For each locus, 2% of the first-round amplicon material (1 μl) was used for 50 μl of reactions, using 0.2 μM (combined final concentration) of an equimolar mix of P7 + UPM_S_4N (5′-gtgactggagttcagacgtgtgctcttccgatctNNNNCTAATACGACTCACTATAGGGC), P7, UPM_S_5N (5′-gtgactggagttcagacgtgtgctcttccgatctNNNNNCTAATACGACTCACTATAGGGC), and P7 + UPM_S_6N (5′-gtgactggagttcagacgtgtgctcttccgatctNNNNNNCTAATACGACTCACTATAGGGC) primers together with 0.2 μM GSPs; other conditions were as for the first round except that amplification was performed for only 20 cycles at an annealing temperature of 55 °C. GSPs used in the second round were OBG_141 (5′-acactctttccctacacgacgctcttccgatctNNNNNCAGGTTCTGGGTTCTGGATGT), OBG_142 (5′-acactctttccctacacgacgctcttccgatctNNNNNNCAGGTTCTGGGTTCTGGATGT), and OBG_143 (5′-acactctttccctacacgacgctcttccgatctNNNNNNNCAGGTTCTGGGTTCTGGATGT) for Tcra; OBG_137 (5-acactctttccctacacgacgctcttccgatctNNNNGGAGTCACATTTCTCAGATCC), OBG_138 (5′-acactctttccctacacgacgctcttccgatctNNNNNGGAGTCACATTTCTCAGATCC), and OBG_139 (5′-acactctttccctacacgacgctcttccgatctNNNNNNGGAGTCACATTTCTCAGATC) for Tcrb. The resulting material was purified with AMPure XP beads (0.65×) and barcoded with NEBNext multiplex oligonucleotides for Illumina. Last, gel purification was used to avoid sequencing fragments shorter than 500 bp in the sequencer. Paired-end sequencing was performed in an Illumina MiSeq instrument at a read length of 300 bp. *Bioinformatic analyses of TCR repertoires*. For the extraction of the sequences, an R pipeline was developed (available upon request). Briefly, unique molecular identifier (UMI) barcodes were used to account for the numbers of cDNA molecules by matching the sequences of UMI, CDR3 region (including the entire J sequence), and a V gene sequence identified from the imgt.org database. Each unique combination of UMI, V, and CDR3 (including the J) was considered to represent a single cDNA molecule but was kept for analysis only if read at least twice and was otherwise discarded. Sequences with UMIs at a distance of one nucleotide and CDR3 sequences at a distance of two nucleotides or less were considered errors; in these instances, only the variant with highest numbers of reads was retained (note, however, that reads not considered after this cutoff are nonetheless contained in the deposited sequence collections to be found at https://www.ncbi.nlm.nih.gov/sra/PRJNA822029. For repertoire analysis, the paired 5′- and 3′- ends of the molecules were not joined but mapped to the V segments separately. The CDR3 region of TCR protein sequences was operationally defined as the sequences occurring between and including the characteristic C-terminal cysteine of V elements and the characteristic phenylalanine residue in J region sequences.

### Statistics and Reproducibility

Two tailed t-tests were used to determine the significance levels of the differences between the means of two independent samples, considering equal or unequal variances as determined by the F-test. For multiple tests, the conservative Bonferroni correction was applied. A P value ≤ 0.05 was considered to be significant. Values are reported as mean ± s.e.m. All replicates for in vitro data are derived from independent experiments. No statistical method was used to predetermine sample size.

### Reporting summary

Further information on research design is available in the Nature Research Reporting Summary linked to this article.

## Supplementary information

Supplementary Information

Description of Additional Supplementary Files

Supplementary Data 1

Supplementary Data 2

Supplementary Data 3

Supplementary Data 4

Supplementary Data 5

Supplementary Data 6

Reporting Summary

## Data Availability

The RNA-seq datasets generated in the present study have been deposited in the National Center for Biotechnology Information’s SRA Archive with accession no. PRJNA822029. Source data are available as Supplementary Data 5 and 6.
