# Supplementary material for: Human thymoma-associated mutation of the GTF2I transcription factor impairs thymic epithelial progenitor differentiation in mice
Source: Commun Biol. 2022 Sep 29;5:1037. doi: 10.1038/s42003-022-04002-7 (PMC9522929; doi:10.1038/s42003-022-04002-7)
Supplement: Supplementary file 1 — Supplementary Information [file 42003_2022_4002_MOESM1_ESM.pdf]

Supplementary Information
for

Human thymoma-associated mutation of the GTF2I transcription factor impairs thymic epithelial progenitor differentiation in mice

Orlando B. Gorgetti, Anja Nusser, Thomas Boehm

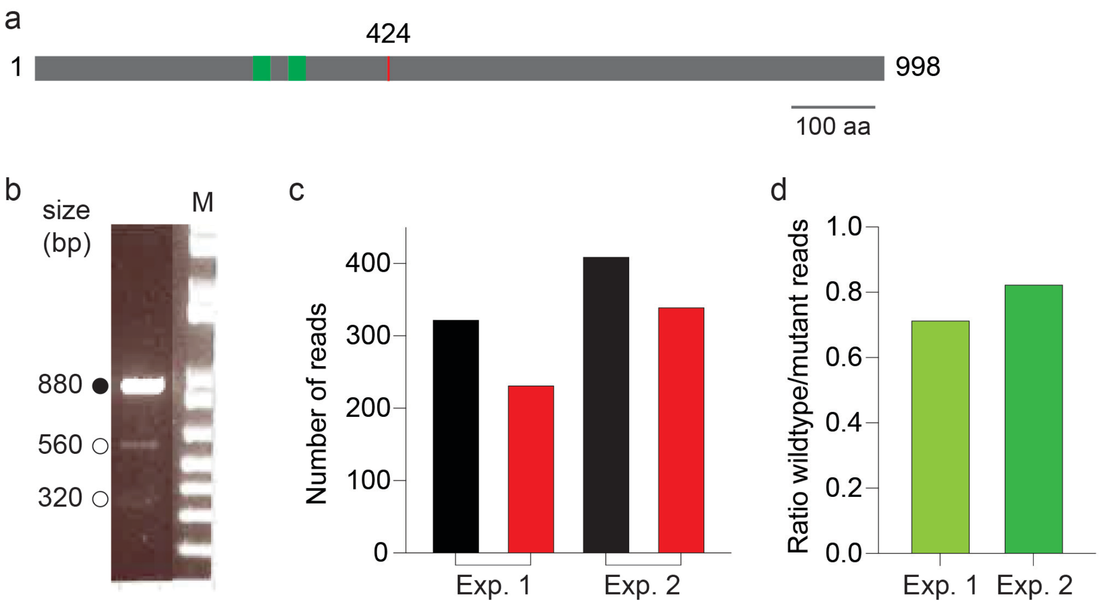

Supplementary Fig. 1 Expression of the mutated form of *Gtf2i* in TECs. a Schematic of the Gtf2i protein. As a result of alternative splicing, two exons are missing in the delta isoform (green boxes; encoding aa 255-274; aa 293-313, respectively. **b** Qualitative assay for mutant transcript expression. An amplicon spanning the mutated site was generated from cDNA of transgenic TECs and digested with *Sph*I (see Methods). The presence of two digestion products (320bp and 560bp) indicates the presence of transcripts emanating from the mutant gene. **c** Quantitative analysis by RNA-seq of wild-type and mutant transcripts in two preparations of TECs. The number of reads covering the wild-type (5´- GGATATTGCTGGCGAAGGAA) or mutant (5´- GGATATTGCATGCGAAGGAA) sequences were determined. **d** Ratio of wild-type and mutant read numbers for the experiments shown in **c**.

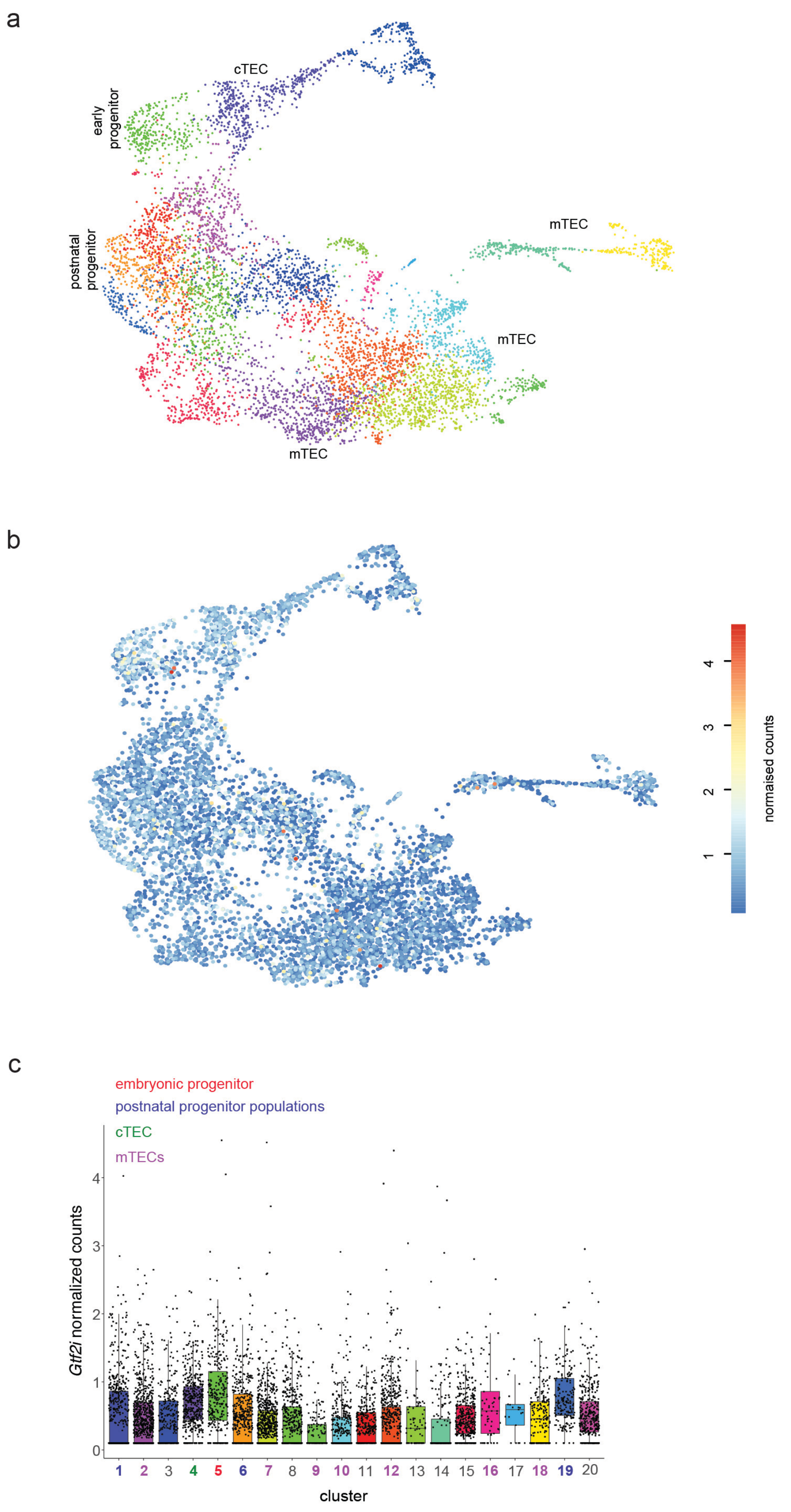
a
cTEC
early progenitor
postnatal progenitor
mTEC
mTEC
mTEC
b
normalised counts
1
2
3
4
c
embryonic progenitor
postnatal progenitor populations
cTEC
mTECs
Gtf2i normalized counts
0
1
2
3
4
1 2 3 4 5 6 7 8 9 10 11 12 13 14 15 16 17 18 19 20
cluster

Supplementary Fig. 2 Expression of *Gtf2i* in TEC subsets of wild-type mice. **a** Uniform manifold approximation and projection (UMAP) representation of transcriptome similarities between 6,959 individual TECs derived from 4-week-old wild-type male (n=2) and female (n=2) mice. The positions of clusters containing early and postnatal bi-potent progenitors, and mature cTEC and mTEC clusters are indicated. Colours mark cells in the identified cell clusters. Modified from[11]. **b** Expression profile of *Gtf2i* in individual TECs; the scale to the right indicates normalised transcript counts. **c** Expression levels of *Gtf2i* in the individual cell clusters depicted in **a** and identified by colour. The clusters contributing to the four relevant TEC compartments discussed here are marked (see also Fig. 3a). The first and third quartiles are marked by the box, the median is denoted by a horizontal line, the boundaries of the whiskers are set at 1.5 times the interquartile range, outliers are indicated as dots outside the boundary of the whiskers.

Supplementary Table 1 Antibodies and staining reagents used in this study.

**Antigen/Reagent**	**Clone**	**Conjugate**	**Supplier**	**Catalogue Number**	**Dilution**
CD4	GK1.5	FITC	BioLegend	100406	1:1000
CD8a	53-6.7	PE	eBioscience	12-0081-85	1:200
CD45	30-F11<br>30-F11	PE Cy7<br>FITC	BioLegend<br>eBioscience	103114<br>11-0451-82	1:2000<br>1:2000
EpCAM	G8.8	APC	BioLegend	118214	1:1000
Keratin 5	rabbit polyclonal	-	Covance	PRB-160P	1:500
Keratin 8	Troma-1	-	produced in house	-	1:200
Ly51	6C3	PE	eBioscience	12-5891-83	1:300
Streptavidin	-	Cy3	Jackson ImmunoResearch	016-160-084	1:1000
Streptavidin	-	PE	eBioscience	12-4317-87	1:1000
UEA-1	-	FITC	Vector Laboratories	FL-1061-5	1:1000
Keratin 18	Ks18.04	biotin	PROGEN	61528	1:10
