# Supplementary material for: Human thymoma-associated mutation of the GTF2I transcription factor impairs thymic epithelial progenitor differentiation in mice
Source: Commun Biol. 2022 Sep 29;5:1037. doi: 10.1038/s42003-022-04002-7 (PMC9522929; doi:10.1038/s42003-022-04002-7)
Supplement: Supplementary file 2 — Description of Additional Supplementary Files [file 42003_2022_4002_MOESM2_ESM.pdf]

Description of Additional Supplementary Files

File name: Supplementary Data 1
Description: Differentially expressed genes in mutant TECs.

File name: Supplementary Data 2
Description: Pathway analysis of upregulated genes in mutant TECs.

File name: Supplementary Data 3
Description: Pathway analysis of downregulated genes in mutant TECs.

File name: Supplementary Data 4
Description: Expression levels of genes implicated in the regulation of apoptosis and WNT signalling.

File name: Supplementary Data 5
Description: Source data for Figure 2.

File name: Supplementary Data 6
Description: Source data for Figure 3.
