# Supplementary material for: Human thymoma-associated mutation of the GTF2I transcription factor impairs thymic epithelial progenitor differentiation in mice
Source: Commun Biol. 2022 Sep 29;5:1037. doi: 10.1038/s42003-022-04002-7 (PMC9522929; doi:10.1038/s42003-022-04002-7)
Supplement: Supplementary file 9 — Reporting Summary [file 42003_2022_4002_MOESM9_ESM.pdf]

nature portfolio

Corresponding author(s): Thomas Boehm

Last updated by author(s): Sep 6, 2022

Reporting Summary

Nature Portfolio wishes to improve the reproducibility of the work that we publish. This form provides structure for consistency and transparency in reporting. For further information on Nature Portfolio policies, see our Editorial Policies and the Editorial Policy Checklist.

Statistics

For all statistical analyses, confirm that the following items are present in the figure legend, table legend, main text, or Methods section.

n/a	Confirmed	
☐	☒	The exact sample size (*n*) for each experimental group/condition, given as a discrete number and unit of measurement
☐	☒	A statement on whether measurements were taken from distinct samples or whether the same sample was measured repeatedly
☐	☒	The statistical test(s) used AND whether they are one- or two-sided<br>*Only common tests should be described solely by name; describe more complex techniques in the Methods section.*
☒	☐	A description of all covariates tested
☐	☒	A description of any assumptions or corrections, such as tests of normality and adjustment for multiple comparisons
☐	☒	A full description of the statistical parameters including central tendency (e.g. means) or other basic estimates (e.g. regression coefficient) AND variation (e.g. standard deviation) or associated estimates of uncertainty (e.g. confidence intervals)
☐	☒	For null hypothesis testing, the test statistic (e.g. *F*, *t*, *r*) with confidence intervals, effect sizes, degrees of freedom and *P* value noted<br>*Give P values as exact values whenever suitable.*
☒	☐	For Bayesian analysis, information on the choice of priors and Markov chain Monte Carlo settings
☒	☐	For hierarchical and complex designs, identification of the appropriate level for tests and full reporting of outcomes
☒	☐	Estimates of effect sizes (e.g. Cohen's *d*, Pearson's *r*), indicating how they were calculated

Our web collection on statistics for biologists contains articles on many of the points above.

Software and code

Policy information about availability of computer code

Data collection	FACS Diva Software v8.0.2.
Data analysis	FlowJo 9.3.1 for flow cytometric analyses, GraphPad Prism 9 for data presentation.

For manuscripts utilizing custom algorithms or software that are central to the research but not yet described in published literature, software must be made available to editors and reviewers. We strongly encourage code deposition in a community repository (e.g. GitHub). See the Nature Portfolio guidelines for submitting code & software for further information.

Data

Policy information about availability of data

All manuscripts must include a data availability statement. This statement should provide the following information, where applicable:

- Accession codes, unique identifiers, or web links for publicly available datasets
- A description of any restrictions on data availability
- For clinical datasets or third party data, please ensure that the statement adheres to our policy

All data needed to evaluate the conclusions in the paper are present in the paper or the Supplementary Materials. The RNA-seq datasets generated in the present study have been deposited in the National Center for Biotechnology Information's SRA Archive with accession no. PRJNA822029 available at https://www.ncbi.nlm.nih.gov/sra/PRJNA822029.

Human research participants

Policy information about studies involving human research participants and Sex and Gender in Research.

Reporting on sex and gender	*Use the terms sex (biological attribute) and gender (shaped by social and cultural circumstances) carefully in order to avoid confusing both terms. Indicate if findings apply to only one sex or gender; describe whether sex and gender were considered in study design whether sex and/or gender was determined based on self-reporting or assigned and methods used. Provide in the source data disaggregated sex and gender data where this information has been collected, and consent has been obtained for sharing of individual-level data; provide overall numbers in this Reporting Summary. Please state if this information has not been collected. Report sex- and gender-based analyses where performed, justify reasons for lack of sex- and gender-based analysis.*
Population characteristics	*Describe the covariate-relevant population characteristics of the human research participants (e.g. age, genotypic information, past and current diagnosis and treatment categories). If you filled out the behavioural & social sciences study design questions and have nothing to add here, write "See above."*
Recruitment	*Describe how participants were recruited. Outline any potential self-selection bias or other biases that may be present and how these are likely to impact results.*
Ethics oversight	*Identify the organization(s) that approved the study protocol.*

Note that full information on the approval of the study protocol must also be provided in the manuscript.

Field-specific reporting

Please select the one below that is the best fit for your research. If you are not sure, read the appropriate sections before making your selection.

☒ Life sciences ☐ Behavioural & social sciences ☐ Ecological, evolutionary & environmental sciences

For a reference copy of the document with all sections, see nature.com/documents/nr-reporting-summary-flat.pdf

Life sciences study design

All studies must disclose on these points even when the disclosure is negative.

Sample size	The sample sizes for each experiment are indicated in the figure legends. Sample sizes were based on our experience and accepted practice in the respective fields, balancing statistic robustness, resource availability, and animal welfare. No statistical methods were used to predetermine sample size.
Data exclusions	No data were excluded.
Replication	Several biological replicates were analysed (see figure legends). The results of these independent experiments were in agreement.
Randomization	Provided the transgenic status, age, and sex of mice matched the experimental requirements, mice were randomly assigned to experimental groups.
Blinding	Blinding was not possible, as the thymus phenotype, i.e. the transgenic status of the respective mouse, is evident from flow cytometry and histological analyses.

Reporting for specific materials, systems and methods

We require information from authors about some types of materials, experimental systems and methods used in many studies. Here, indicate whether each material, system or method listed is relevant to your study. If you are not sure if a list item applies to your research, read the appropriate section before selecting a response.

Materials & experimental systems

n/a	Involved in the study
☐	☒ Antibodies
☒	☐ Eukaryotic cell lines
☒	☐ Palaeontology and archaeology
☐	☒ Animals and other organisms
☒	☐ Clinical data
☒	☐ Dual use research of concern

Methods

n/a	Involved in the study
☒	☐ ChIP-seq
☐	☒ Flow cytometry
☒	☐ MRI-based neuroimaging

Antibodies

Antibodies used

α-CD4 (clone GK1.5; FITC conjugate; Cat#100406, BioLegend); α-CD8a (clone 53-6.7; FITC conjugate; Cat#11-0081-82, eBioscience); α-CD8a (clone 53-6.7; APC conjugate; Cat#17-0081-82, eBioscience); α-CD31 (clone MEC13.3; FITC conjugate; Cat#102506, BioLegend); α-CD45 (clone 30-F11; PE-Cy7 conjugate; Cat#103114, BioLegend); α-EpCAM (clone G8.8; APC conjugate; Cat#118214, BioLegend); α-Keratin5 (rabbit polyclonal; Cat#PRB-160P, Covance); α-Keratin-8 (clone Troma-1; produced in-house); α-Ly51 (clone 6C3; PE conjugate; Cat#12-5891-83, eBioscience); Streptavidin (eFluor450 conjugate; Cat#48-4317-82, eBioscience; Streptavidin (Cy3 conjugate; Cat#016-160-084, Jackson ImmunoResearch); UEA-1 (FITC conjugate; Cat# FL-1061-5, Vector Laboratories); Keratin 18 Ks18.04 (biotin-conjugate; Cat# 61528,PROGEN); mCardinal (RFP) rabbit polyclonal (Cat# R10367, ThermoFisher Scientific), MHC2 rat (M5/114.15.2; FITC conjugate; Cat#107606; BioLegend); rabbit IgG (H+L) (goat polyclonal; Alexa Fluor 633 conjugate; Cat#A-21070; Invitrogen Molecular Probes); rat IgG (H+L) (donkey polyclonal; Cy3 conjugate; Cat#712-166-153; Jackson ImmunoResearch).

Validation

Validation data are available from the manufacturers´ websites: see information associated with catalog numbers; https://www.biolegend.com/; https://www.thermofisher.com/; https://www.bioz.com/result/anti%20keratin%205/product/Covance; https://www.jacksonimmuno.com/; https://vectorlabs.com/

Animals and other research organisms

Policy information about studies involving animals; ARRIVE guidelines recommended for reporting animal research, and Sex and Gender in Research

Laboratory animals

C57BL/6 mice are maintained in the Max Planck Institute of Immunobiology and Epigenetics. The Foxn1:Gtf2iL384H transgene was created by inserting a cDNA fragment corresponding to nucleotides 1 to 4300 in GenBank accession number AK147201.1 as a NotI fragment into pAHB14 (ref. 17); the TG>AT mutation was inserted into the wild-type cDNA sequence by site-directed mutagenesis according to standard procedures, and verified by Sanger sequencing. Note that L384 is the equivalent of L424 in the human sequence in the delta isoform of Gtf2i. The mutated site creates a recognition sequence for the SphI restriction endonuclease (5´-GCATGC-3´; mutant nucleotides in bold). Transgenic mice were generated on an FVB/N background (FVB/N-tg (Gtf2iL384H)1Tbo/Mpie) and subsequently backcrossed to a C57BL/6J background. Mice were kept in the animal facility of the Max Planck Institute of Immunobiology and Epigenetics under specific pathogen-free conditions.

Wild animals

n/a

Reporting on sex

Mouse of both sexes were used. No differences in phenotypes were observed between male and female mice

Field-collected samples

n/a

Ethics oversight

All animal experiments were approved by the institute’s review committee and conducted under license from and approved by the local government (Regierungspräsidium Freiburg) (license 35-9185.81/G-15/36).

Note that full information on the approval of the study protocol must also be provided in the manuscript.

Flow Cytometry

Plots

Confirm that:

☒ The axis labels state the marker and fluorochrome used (e.g. CD4-FITC).

☒ The axis scales are clearly visible. Include numbers along axes only for bottom left plot of group (a 'group' is an analysis of identical markers).

☒ All plots are contour plots with outliers or pseudocolor plots.

☒ A numerical value for number of cells or percentage (with statistics) is provided.

Methodology

Sample preparation

To generate single cell suspensions for analytical and preparative flow cytometry of TECs, the procedures described by

Sample preparation	Nagakubo et al., Sci. Rep. 2017 and Rode et al., J. Immunol. 2015 were followed. Note that the enzymatic cocktail required to liberate thymic epithelial cells destroys the extracellular domains of CD4 and CD8 surface markers (but not that of the CD45 molecule); hence, when analysis of thymocyte subsets was desired, thymocyte suspensions were prepared in parallel by mechanical liberation, achieved by gently pressing thymic lobes through 40 µm sieves. To isolate thymic mesenchymal and endothelial cells, the cell suspension of total thymocytes was depleted of CD45+ cells; the EpCAM–CD45– cell population was stained with Ly51 and CD31 to purify EpCAM–CD31–Ly51+ mesenchymal and EpCAM–CD31+Ly51– endothelial cells.
Instrument	BD Fortessa II; from Dako Cytomation-Beckman Coulter BD Fortessa II; MoFlow; both from Dako Cytomation-Beckman Coulter
Software	BD FACSDiva for data collection, Summit for sorting TECs, FlowJo for data analysis, statistical analysis with Graphpad Prism
Cell population abundance	Purity was determined by running a purity check of the sorted populations after the sort was completed.
Gating strategy	All samples were initially gated using forward and side scatter to identify events corresponding to cells, doublets are excluded by gating on single cells using forward scatter height vs. area, alive cells were selected by negativity for the viability dye Fluoro Gold, the follow gating steps are according to the relevant surface molecules.

☒ Tick this box to confirm that a figure exemplifying the gating strategy is provided in the Supplementary Information.
